# Neuritis and Myelitis and the Forms of Paralysis and Pseudo-Paralysis Following Labor1Read before the College of Physicians, Philadelphia, March 1, 1893.

**Published:** 1893-07

**Authors:** Charles K. Mills

**Affiliations:** Philadelphia


### Abstract.

### NEURITIS AND MYELITIS AND THE FORMS OF PARALYSIS AND PSEUDO-PARALYSIS FOLLOWING LABOR.[1]

By CHARLES K. MILLS, M. D., Philadelphia.

NEURAL and spinal affections of puerperal origin, although not entirely neglected, have received but little attention in the text-books and journals. Imbert-Gourbeyre[2] refers briefly to the occurrence of paralyses from trauma during labor, in a monograph on Puerperal Paralyses, giving several cases. One of these, from Rademacher, was an incomplete and painful paraplegia coming on at the end of a long and difficult labor, and cured in eight days chiefly by friction. Another patient, thirty-six years old, during her third labor was paralyzed in both extremities, and recovered in a few months. Salvat is recited as reporting the case of a woman treated by him for vesico-vaginal fistula, with paraplegia produced by the long stay of the head of the fetus in the inferior strait. Another patient, thirty-two years old, during her fourth labor, which was prolonged and the delivery by forceps, suffered great pain in the loins, accompanied by feebleness and swelling of the legs. The feebleness increased to paraplegia, and she had lancinating pains, paresthesia and cramps in the limbs. I have cited these cases in a paper on Lesions of the Sacral and Lumbar Plexuses,[3] in which I have also reported two cases of sacral neuritis, kindly furnished me by Dr. Howard A. Kelly, of the Johns Hopkins Hospital. The first patient had passed through a difficult instrumental confinement some twelve years before coming under observation. She visited many prominent gynecologists, and underwent a number of operations, the last being the removal of two large tubes and ovaries. She was relieved of menstrual exacerbations, but still suffered great pain in the pelvis, for which she was receiving galvanism, massage, and antilithic remedies. A careful examination showed that the uterus and its surroundings were perfectly free from disease. On making a careful rectal examination, however, outlining the sacro-sciatic ligament and pyriformis muscles, and carefully palpating the roots of the great sciatic nerve, upon

1. Read before the College of Physicians, Philadelphia, March 1, 1893.

2. Imbert-Gourbeyre: Mem. de l'Acad. Imp. de Med., tome xxv., 1861.

3. Mills, *Med. News*, June 15, 1889.

touching one cord she gave a sudden scream, at the same time doubling up her leg and jerking her body in the bed. Here, directly over the roots of the left sciatic nerve,—the left sacral plexus,—was the only diseased area which could be detected in the pelvis. All subsequent treatment was directed to this condition. The second patient had constant pelvic pain, which she described as soreness, located at the "back of the womb." She had been for several years, since the birth of her last child, under the care of gynecologists, who had not been able to give her any relief whatever. It was found by exploration that the only point of tenderness in the pelvis was at the roots of the sciatic nerve, and here she at once located all her pain when the doctor introduced his finger into the rectum and made pressure on the nerve trunks. The case was one of neuritis. As Dr. Kelly remarks in the communication sending notes of these cases, they teach the value of exploration of the pelvis outside of the uterus and its annexes.

Ramsbotham[1] speaks of paralysis of both legs of varying degree occasionally happening after labor ; more frequently when the process has been tedious and painful, but sometimes when it has been of ordinary duration, or even of unusual rapidity. He is evidently referring to the same classes of cases of which we are treating in the present paper. "It is not attended with cerebral affection," he says, "but is dependent on pressure which the muscles and nerves have sustained during the passage of the child's head through the pelvis. There is pain and numbness, both within the cavity and around the hip, and an inability to move the limb with freedom. It generally disappears by degrees within a few days ; at other times it continues beyond the period the patient commonly remains in bed, and compels her when she rises from it to use a stick or a crutch. Fomentations in the first instance, and afterward stimulating embrocations, a douche or shower bath, tonic medicines and gentle movement of the limb, will offer us the best chances of success. Hemiplegia, indeed, may appear after delivery as well as at other times ; but there will then be particular symptoms, independent of those connected with the local affection, which are too well known to require mention from us here."

Cazeaux and Tarnier,[2] in the American edition of their treatise, translated by Hess, under Lesions of Innervation, direct attention

1. Ramsbotham : The Principles and Practice of Obstetric Medicine and Surgery. Am. ed., 1865.

2. Cazeau and Tarnier : Theory and Practice of Obstetrics. Eighth Am. ed., edited and revised by R. J. Hess, M. D. 1887.

to the various forms of paraplegia occurring during pregnancy or labor, most of which they attribute to reflex causes. Lloyd[1] considers some aspects of the subject in a contribution to Hirst's American System of Obstetrics.

Winckel[2] introduces a brief chapter on the Neuralgias and Paralyses of the Lower Limbs, with a paragraph which is comprehensive in its presentation of the etiology of these affections :

The puerperal neuroses of the lower limbs are located chiefly in the nerve trunks, more rarely in the nerve centers, and generally owe their development to parturition. Injurious pressure is effected by a large, hard, child's head, unfavorable in presentation in a small pelvis. Vigorous compression may result in complete interruption of conduction at the compressed spot. This occurs in instrumental aid during labor, inasmuch as the rim of the blades of the forceps may produce severe contusion of the sacral plexus on forced closure as well as during extraction. The thick nerves are also compressed not infrequently by a pelvic exudation, or small extravasations from the neighboring parts extending to the sheaths of the nerves, or hyperemia, or edema of the neurilemma appear spontaneously. We have already mentioned that parametritis may give rise to such neuralgias. The location of the affection is in the external and middle cutaneous, obturator, or sciatic nerves. The two latter nerves are especially apt to suffer during labor. Even the slight pelvic exudations, for example, one resulting from phlebitis, may make a nerve trunk incapable of conduction without compressing the entire trunk. (Leyden.) Injuries of the vagina, with subsequent severe cicatricial contraction, may also exercise traction and pressure on individual nerve trunks of the pelvis minor, and hyperesthesia and motor disturbances may thus be produced.

References to this subject are also to be found in some of the neurological text-books, but it is not in these treated of systematically.

To Dr. Anna M. Fullerton, of the Woman's Hospital of Philadelphia, who, in her large obstetrical experience, has seen a number of instances of paralysis and pseudo-paralysis during the puerperium, I am indebted for the notes of some cases. In a letter accompanying these notes, she says that partial paralysis is, in general, short-lived, being limited generally to two or three days. She has seen paralyses of the rectum and sphincter from long-continued pressure of the head upon the floor of the pelvis, which sometimes persisted for a week or ten days, and which was only overcome by faradism and time ; also vesical paralysis, which

1. Lloyd : Hirst's System of Obstetrics, by American authors. Vol. II.

2. Winckel : A Text-Book of Obstetrics. Translated by J. Clifton Edgar, A. M., M. D., 1890.

has sometimes lasted up to the sixth, seventh, or twelfth day after delivery. According to Dr. Fullerton, cases of contracted pelvis, which, because of a narrow inlet, have resisted the engagement or descent of the head, and cases in which for any cause there has been impaction of the fetal head, have been those most commonly resulting in paralysis.

. . . . . . . . . . . .

The most valuable recent paper on puerperal paralysis of peripheral origin is that of Hünermann,[1] assistant in the clinic of Gusserow, at Berlin, on Paralysis in the Region of the Sciatic Nerve Following Labor. On examination of this paper, which I have obtained quite recently, I find that Hünermann has reasoned with reference to the production of the most common type of these palsies in much the same manner as I had already presented the subject in the arena at the Philadelphia Hospital, and to my ward classes. He carefully traverses the literature of the subject, and his paper can be consulted with advantage for French and German references. Among the writers to whom he refers are: von Renz, Koeppen, von Leyden, Kast, Möbius, von Basedow, Gerber, Breisky, Litzmann, Kehrer, Dorion, Bianchi, Lefebre, Brivois, Guinon and Parmentier, and Handford.

. . . . . . . . . . . . . .

The affections to which attention has been directed in the paper is discussed under five heads:

(1) Traumatic paralysis of the peroneal type, usually associated with severe neuritis.

(2) Sacral and sacro-distal neuritis, sometimes accompanied by a pseudo-paralysis, and often maintained or aggravated by disease and displacements of the pelvic organs and tissues.

(3) Puerperal neuritis, local or multiple, and due to septic or other infection.

(4) The neuritis, paralysis, and pseudo-paralyses of phlebitis, and phlegmasia alba dolens, which are often septic, but have special features.

(5) Puerperal myelitis occurring under the same conditions as the forms of septic and infectious neuritis.

Brief reference is made to forms of hysterical and reflex paralysis, which must be diagnosticated from the affection under consideration. Cases illustrative of each of the classes mentioned are presented, the first class, however, receiving the fullest discussion. —*University Medical Magazine.*

1. Hünermann: *Archiv. für Gynäkologie*, Berlin, 1892, Vol. XLII., Part III.

